# Association of Lifestyle Intervention With Risk for Cardiovascular Events Differs by Level of Glycated Hemoglobin

**DOI:** 10.1210/clinem/dgad674

**Published:** 2023-11-18

**Authors:** Michael P Bancks, Scott J Pilla, Ashok Balasubramanyam, Hsin-Chieh Yeh, Karen C Johnson, Joseph Rigdon, Lynne E Wagenknecht, Mark A Espeland

**Affiliations:** Department of Epidemiology and Prevention, Wake Forest University School of Medicine, Winston-Salem, NC 27157, USA; Department of Medicine, Johns Hopkins School of Medicine, Baltimore, MD 21205, USA; Department of Medicine, Baylor College of Medicine, Houston, TX 77030, USA; Department of Medicine, Johns Hopkins School of Medicine, Baltimore, MD 21205, USA; Department of Preventive Medicine, University of Tennessee Health Science Center, Memphis, TN 38163, USA; Department of Biostatistics and Data Science, Wake Forest University School of Medicine, Winston-Salem, NC 27157, USA; Department of Epidemiology and Prevention, Wake Forest University School of Medicine, Winston-Salem, NC 27157, USA; Department of Biostatistics and Data Science, Wake Forest University School of Medicine, Winston-Salem, NC 27157, USA; Department of Internal Medicine, Wake Forest University School of Medicine, Winston-Salem, NC 27157, USA

**Keywords:** cardiovascular disease, HbA1c, intensive lifestyle intervention, overweight/obesity, type 2 diabetes mellitus

## Abstract

**Purpose:**

We reevaluated the Action for Health in Diabetes (Look AHEAD) intensive lifestyle intervention (ILI) to assess whether the effect of ILI on cardiovascular disease (CVD) prevention differed by baseline glycated hemoglobin (HbA1c).

**Methods:**

Look AHEAD randomized 5145 adults, aged 45 to 76 years with type 2 diabetes and overweight/obesity to ILI or a diabetes support and education (DSE) control group for a median of 9.6 years. ILI focused on achieving weight loss through decreased caloric intake and increased physical activity. We assessed the parent trial's primary composite CVD outcome. We evaluated additive and multiplicative heterogeneity of the intervention on CVD risk by baseline HbA1c.

**Results:**

Mean baseline HbA1c was 7.3% (SD 1.2) and ranged from 4.4% (quintile 1) to 14.5% (quintile 5). We observed additive and multiplicative heterogeneity of the association between ILI and CVD (all *P* < .001) by baseline HbA1c. Randomization to ILI was associated with lower CVD risk for HbA1c quintiles 1 [hazard ratio (HR): 0.68, 95% confidence interval (CI): 0.53, 0.88] and 2 (HR: 0.80, 95% CI: 0.66, 0.96) and associated with higher CVD risk for HbA1c quintile 5 (HR: 1.27, 95% CI: 1.02, 1.58), compared to DSE.

**Conclusion:**

Among adults with type 2 diabetes and overweight/obesity, randomization to a lifestyle intervention was differentially associated with CVD risk by baseline HbA1c such that it was associated with lower risk at lower HbA1c levels and higher risk at higher HbA1c levels. There is a critical need to develop and tailor lifestyle interventions to be successful for individuals with type 2 diabetes and high HbA1c.

Action for Health in Diabetes (Look AHEAD) was a randomized controlled trial evaluating the cardiovascular effects of intensive lifestyle intervention (ILI) designed to achieve and maintain weight loss compared to diabetes support and education (DSE). Over a decade of follow-up, ILI was not associated with lower cardiovascular disease (CVD) morbidity or mortality compared to DSE across the full cohort [hazard ratio (HR) ILI to DSE: 0.95, 95% confidence interval (CI): 0.83, 1.09] (1). Post hoc analyses suggest that ILI may provide benefit compared to DSE for cardiovascular outcomes depending on prior CVD history and baseline predicted risk for CVD (1-4). We previously characterized 4 distinct type 2 diabetes subgroups in Look AHEAD and observed that the association of ILI on risk for the primary composite CVD outcome and 2 of 3 secondary composite CVD outcomes differed according to these type 2 diabetes subgroups (5). Notably, randomization to ILI was associated with 1.85 times (95% CI: 1.32, 2.61) higher risk for CVD among a type 2 diabetes subgroup characterized by high baseline level of glycated hemoglobin (HbA1c), while ILI was not associated with CVD risk among the remaining 3 type 2 diabetes subgroups. A combination of clinical characteristics with a statistical clustering technique were used to determine the type 2 diabetes subgroups based on a multifactorial profile, and we could not infer directly that high HbA1c modified the effect of lifestyle intervention on CVD prevention. Behavioral lifestyle change is a foundational first-line strategy in management of type 2 diabetes. It is critical to know whether the effect of the Look AHEAD lifestyle intervention differed in CVD prevention across the spectrum of baseline HbA1c, particularly for individuals with high HbA1c and corresponding high risk for CVD (6), so that we can identify individuals who may need tailored interventions for successful CVD prevention. Our primary objective was to assess whether baseline HbA1c level alone may impact the effect of an intensive lifestyle intervention on CVD prevention and secondary objectives were to explore potential explanations for any observed differential associations.

## Methods

This is a secondary analysis of the Look AHEAD trial, for which the study design and methods have been described (7). Look AHEAD was a randomized controlled trial among individuals with type 2 diabetes and overweight or obesity. From August 2001 through April 2004, 5145 participants were recruited at 16 clinical sites across the United States. Eligibility criteria for Look AHEAD included being age 45 to 76 years; type 2 diabetes verified by the use of glucose-lowering medication, a physician's report, or elevated fasting glucose levels; and a body mass index of ≥25.0 kg/m^2^ (≥27.0 kg/m^2^ if taking insulin). Individuals were excluded from study participation for any of the following at their initial screening examination: HbA1c of >11% (>97 mmol/mol); systolic blood pressure ≥ 160 mm Hg; diastolic blood pressure ≥ 100 mm Hg; triglyceride level ≥ 6.77 mmol/L; inability to complete a valid maximal exercise test; and lacking an established relationship with a primary care provider. At the time of randomization, 40 individuals (26 DSE, 14 ILI) had an HbA1c > 11% (median 65 days from screening visit to randomization; interquartile range days: 46, 90). Participants provided informed consent and local Institutional Review Boards approved the study protocol.

### Study Intervention

Study participants were randomly assigned 1:1 within the clinical site to receive either a multidomain ILI or a comparison condition of diabetes support and education (DSE) (7-9). The intervention aimed to achieve and maintain participant weight loss of ≥7% via decreased caloric intake (1200 to 1800 kcal per day) and increased physical activity (≥175 minutes of moderate-intensity per week). ILI participants received group and individual counseling sessions, occurring weekly during the first 6 months, with decreasing frequency over the course of the trial. Participants who were randomized to DSE were invited to 3 group educational sessions each year in the first 4 years of the trial, reduced thereafter to 1 annual session. All adjustments to medications were made by the participant's health care provider, except for temporary changes in glucose-lowering medications made by study staff to reduce the risk of hypoglycemia associated with weight loss in the ILI group. While participants were not masked to intervention allocation, nonintervention study staff and investigators were masked to intervention status. The intervention was terminated after 9.6 years median follow-up and participants continued to be followed.

### Clinical Assessments

At baseline and annual study clinic visits, staff members certified in assessment procedures measured height, weight, waist circumference, and blood pressure. With participants in light clothing, height and weight were measured in duplicate using a standard stadiometer and digital scale, respectively. Resting seated blood pressure was measured in duplicate with a Dinamap Monitor Pro100 automated device. Fasting blood was drawn and aliquoted, and samples were shipped on dry ice to the Look AHEAD Central Biochemistry Laboratory (Northwest Lipid Research Laboratories, Seattle, WA, USA) where analyses were performed. HbA1c was measured using a dedicated ion-exchange high-performance liquid chromatography instrument (Bio-Rad Variant II) (10). Blood was drawn at the screening visit and randomization visit, and HbA1c at the time of randomization was used for analysis. Interviewer-administered questionnaires were used to collect information on participant medical history, employment, education, family income, prior pregnancies, smoking, prescription medications, alcohol use, and family medical history.

### Study Endpoints

We assessed the parent trial's prespecified primary and secondary endpoints from randomization through September 14, 2012, when the intervention was stopped (1, 7). The original primary endpoint was the first occurrence of a composite cardiovascular outcome, which included death from cardiovascular causes, nonfatal myocardial infarction, and nonfatal stroke. The Data and Safety Monitoring Board assessed the study progress at the 2-year mark and reported the primary-event rate in the control group was lower than expected and adjudicated hospitalization for angina was added to the primary composite outcome that is used for the current analysis (11). Three secondary composite cardiovascular outcomes were also assessed: (1) death from cardiovascular causes, nonfatal myocardial infarction, or nonfatal stroke; (2) death from any cause, myocardial infarction, stroke, or hospitalization for angina; and (3) death from any cause, myocardial infarction, stroke, hospitalization for angina, coronary-artery bypass grafting, percutaneous coronary intervention, hospitalization for heart failure, carotid endarterectomy, or peripheral vascular disease. Staff masked to intervention assignment asked participants about all medical events and hospitalizations at annual visits and 6-month phone calls and conducted searches of national databases on deaths. Hospital records were obtained for potential CVD events, and trained adjudicators masked to intervention assignment adjudicated potential events using standardized criteria (1).

### Statistical Analysis

There is a known relationship of high HbA1c with higher CVD risk, and our goal was not to assess the main effect of HbA1c on CVD risk but rather to assess whether baseline HbA1c level modifies the relationship between ILI and CVD risk (6, 12). We created quintiles of baseline HbA1c and summarized randomization allocation, demographic, and clinical characteristics by HbA1c quintile. We assessed whether randomization to ILI compared to DSE was associated with differential risk for the primary and secondary study endpoints according to continuous baseline HbA1c level and HbA1c quintile. To do so, we used Cox proportional hazards regression and included main effects for intervention arm, study site and history of CVD because these were part of stratified randomization, and baseline HbA1c and a product term of the intervention arm and baseline HbA1c to estimate HRs and 95% confidence limits. Characteristics that may confound the association between HbA1c and CVD risk were not included for adjustment in the primary analysis because we are not inferring a causal interaction between HbA1c and lifestyle intervention on CVD risk (13). We estimated risk differences using Poisson regression with the robust variances with similar adjustment (14). We started with assessment of the primary outcome and observed evidence for effect modification of the intervention on CVD risk by baseline HbA1c, which supported the use of estimating the intervention effect within HbA1c quintile. We assessed if the statistical interaction between intervention arm and continuous HbA1c was nonlinear with a quadratic term for HbA1c. We performed primary analyses according to the intention-to-treat principle and sensitivity analyses that included adjustment for age at randomization, sex, race/ethnicity, HbA1c, diabetes duration, insulin medication use, body mass index, cardiovascular history, clinical site, education, smoking status, low-density lipoprotein cholesterol, systolic blood pressure, blood pressure lowering medication, and lipid-lowering medication use.

We performed exploratory analyses of potential contributors to the relationship between baseline HbA1c and the efficacy of ILI vs DSE by describing longitudinal between-arm differences in weight, HbA1c, and diabetes and cardiovascular medication use, stratified by baseline HbA1c quintile. We tested whether there were significant between-arm differences in these factors using generalized linear regression and generalized estimating equations. We assessed sensitivity of our results to differences by baseline HbA1c in achieving the on-intervention weight loss goal of 7% at year 2 of the intervention, respectively, based on prior results suggesting an overall CVD benefit from weight loss (15). We chose to focus on weight loss at year 2 to assess sustainability of weight loss, as peak mean weight loss was achieved in Look AHEAD at year 1 of the intervention with moderate mean weight regain after the initial loss (16). In this weight-loss sensitivity analysis, we included adjustment for clinical and demographic characteristics noted earlier and inverse probability of attrition weighting to account for potential selection bias from cohort attrition related to intervention arm, HbA1c, and CVD risk over the first 2 years. We also assessed whether excluding individuals who underwent bariatric surgery during the intervention phase and whether competing risk of noncardiovascular death impacted our primary findings. All analyses by baseline HbA1c are post hoc, not prespecified in the original Look AHEAD protocol. We used a 2-sided alpha of .05 and SAS version 9.4 (SAS Institute, Cary, NC, USA) for analysis.

## Results

All 5145 Look AHEAD participants had a measurement of HbA1c at randomization. HbA1c at the time of randomization ranged from 4.4% to 14.5% with a median of 7.1% (mean = 7.3%) and interquartile range of 6.4% to 7.9% (median 54 mmol/mol; interquartile range: 46 to 63 mmol/mol). HbA1c distribution did not differ by intervention arm, and equal allocation of randomization arm was maintained within HbA1c quintile. Multiple demographic-, socioeconomic-, diabetes-, and CVD-related characteristics were associated with higher baseline HbA1c but did not differ by intervention arm overall or within HbA1c quintile (Table 1). Younger age and higher body mass index, blood pressure, and cholesterol were associated with higher HbA1c. There was greater prior CVD, family history of diabetes, and use of diabetes medications (insulin and metformin) at baseline with higher HbA1c. HbA1c quintile (Q) 1 and Q5 had the lowest blood pressure- and cholesterol-lowering medication use with Q 2 to 4 having higher use. The most prevalently used blood pressure- and cholesterol-lowering medications at baseline were angiotensin converting enzyme (ACE) inhibitors (44%) and statins (44%), respectively.

**Table 1.** Participant characteristics at randomization according to quintile of HbA1c (Look AHEAD)

Characteristics*^a^*	Quintile 1		Quintile 2		Quintile 3		Quintile 4		Quintile 5	
HbA1c %, range	4.4-6.3		6.4-6.8		6.9-7.3		7.3-8.1		8.2-14.5	
Intervention arm	ILI	DSE	ILI	DSE	ILI	DSE	ILI	DSE	ILI	DSE
n (% total of quintile)	591 (53)	524 (47)	497 (49)	515 (51)	476 (48)	516 (52)	516 (51)	500 (49)	490 (49)	520 (51)
HbA1c %, mean (SD)	6.0 (0.3)	5.9 (0.3)	6.6 (0.1)	6.6 (0.1)	7.1 (0.1)	7.1 (0.1)	7.7 (0.2)	7.7 (0.2)	9.1 (0.8)	9.2 (0.9)
Female, n (%)	332 (56)	316 (60)	298 (60)	312 (61)	284 (60)	311 (60)	310 (60)	293 (59)	302 (62)	305 (59)
Age, years (SD)	59 (7)	59 (7)	59 (7)	60 (7)	59 (7)	59 (7)	58 (7)	59 (7)	57 (7)	57 (7)
Race/ethnicity, n (%)										
White	397 (67)	351 (67)	343 (69)	350 (68)	301 (63)	341 (66)	318 (62)	312 (62)	262 (53)	277 (53)
Black	78 (13)	67 (13)	76 (15)	67 (13)	74 (16)	74 (14)	82 (16)	89 (18)	90 (18)	107 (21)
Native American/American Indian/Alaska Native	31 (5)	23 (4)	19 (4)	17 (3)	24 (5)	33 (6)	21 (4)	21 (4)	35 (7)	34 (7)
Asian/Pacific Islander	10 (2)	3 (1)	3 (1)	7 (1)	5 (1)	4 (1)	6 (1)	1 (0)	5 (1)	6 (1)
Hispanic	62 (10)	68 (13)	49 (10)	61 (12)	60 (13)	55 (11)	77 (15)	66 (13)	92 (19)	90 (17)
Other/multiple	13 (2)	12 (2)	7 (1)	13 (3)	12 (3)	9 (2)	12 (2)	11 (2)	6 (1)	6 (1)
Education >16 years, n (%)	279 (47)	221 (42)	215 (43)	228 (44)	195 (41)	215 (42)	202 (39)	199 (40)	192 (39)	191 (37)
Current smoker, n (%)	23 (4)	17 (3)	17 (3)	17 (3)	22 (5)	23 (4)	21 (4)	25 (5)	34 (7)	28 (5)
No regular alcohol consumption, n (%)	382 (65)	354 (68)	336 (68)	338 (66)	320 (67)	318 (62)	370 (72)	360 (72)	343 (70)	374 (72)
Exercise test fitness, Mets, median (IQR)	7.4 (6.1, 9.0)	7.1 (5.8, 8.5)	7.0 (5.8, 8.5)	7.0 (5.8, 8.5)	7.0 (5.8, 8.4)	7.0 (5.7, 8.5)	6.8 (5.6, 8.0)	6.7 (5.6, 8.0)	6.6 (5.6, 8)	6.7 (5.7, 8.0)
Physical activity,*^b^* kcal/week, median (IQR)	420 (84, 1254)	560 (140, 1300)	502 (92, 176)	448 (112, 1236)	616 (196, 1344)	560 (168, 1344)	462 (112, 1064)	574 (140, 1216)	504 (112, 1236)	364 (84, 908)
Duration of diabetes, years, median (IQR)	2 (1, 6)	3 (1, 6)	4 (1, 8)	4 (2, 8)	5 (2, 10)	5 (3, 9)	6 (3, 11)	6 (3, 11)	7 (4, 12)	7 (4, 12)
Family history of diabetes, n (%)	340 (58)	353 (67)	323 (65)	325 (63)	303 (64)	338 (66)	336 (65)	325 (65)	321 (66)	383 (74)
Diabetes medication—any use, n (%)	444 (75)	406 (77)	426 (86)	440 (85)	419 (88)	458 (89)	480 (93)	456 (91)	473 (97)	502 (97)
Metformin use, n (%)	307 (52)	259 (49)	303 (61)	310 (60)	304 (64)	307 (59)	345 (67)	329 (66)	313 (64)	343 (66)
Sulfonylurea use, n (%)	192 (32)	162 (31)	198 (40)	203 (39)	226 (47)	262 (51)	277 (54)	253 (51)	272 (56)	287 (55)
Thiazolidinedione use, n (%)	123 (21)	134 (26)	117 (24)	131 (25)	125 (26)	134 (26)	132 (26)	144 (29)	145 (30)	142 (27)
Insulin use, n (%)	23 (4)	28 (5)	47 (9)	46 (9)	67 (14)	81 (16)	100 (19)	94 (19)	148 (30)	161 (31)
Number of diabetes medications, mean (SD)	1.1 (0.9)	1.1 (0.9)	1.4 (0.9)	1.3 (0.9)	1.6 (1.0)	1.5 (1.0)	1.7 (0.9)	1.7 (1.0)	1.9 (0.9)	1.9 (1.0)
Body mass index, kg/m^2^ (SD)	35 (6)	36 (5)	36 (6)	36 (6)	36 (6)	36 (6)	36 (6)	36 (6)	37 (6)	37 (6)
SBP, mm Hg (SD)	126 (17)	127 (17)	128 (17)	130 (17)	128 (17)	130 (18)	130 (18)	129 (17)	130 (18)	131 (17)
DBP, mm Hg (SD)	70 (9)	69 (9)	70 (10)	70 (10)	70 (9)	71 (10)	70 (10)	70 (10)	71 (10)	72 (10)
Hypertension medication use, n (%)	430 (73)	385 (73)	383 (77)	367 (71)	367 (77)	378 (73)	386 (75)	377 (75)	348 (71)	370 (71)
LDL cholesterol, mg/dL (SD)	112 (31)	112 (29)	110 (31)	111 (31)	112 (31)	111 (31)	112 (30)	111 (31)	117 (33)	117 (33)
HDL cholesterol, mg/dL (SD)	44 (12)	45 (12)	44 (12)	44 (12)	44 (12)	43 (11)	43 (12)	43 (12)	42 (11)	42 (12)
Cholesterol medication use, n (%)	285 (48)	259 (49)	264 (53)	278 (54)	258 (54)	273 (53)	261 (51)	277 (55)	238 (49)	233 (45)
Prior history of CVD, n (%)	66 (11)	59 (11)	75 (15)	59 (11)	63 (13)	74 (14)	87 (17)	81 (16)	74 (15)	74 (14)

Abbreviations: BMI, body mass index; CVD, cardiovascular disease; DBP, diastolic blood pressure; DSE, diabetes support and education; HbA1c, glycated hemoglobin; HDL, high-density lipoprotein; ILI, intensive lifestyle intervention; IQR, interquartile range; kcal, kilo-calories; LDL, low-density lipoprotein; Mets, metabolic equivalents; mm Hg, millimeters mercury; SBP, systolic blood pressure.

^*a*^Values are means (SD) or counts (column percentage) except when noted for “range” and “median (IQR).”

^*b*^Paffenbarger physical activity questionnaire data was missing for 53% of the sample, similar missing data percentage by randomization arm within HbA1c quintile.

### Association of Randomized Intervention With Cardiovascular Risk by Baseline HbA1c

**Figure 1.**
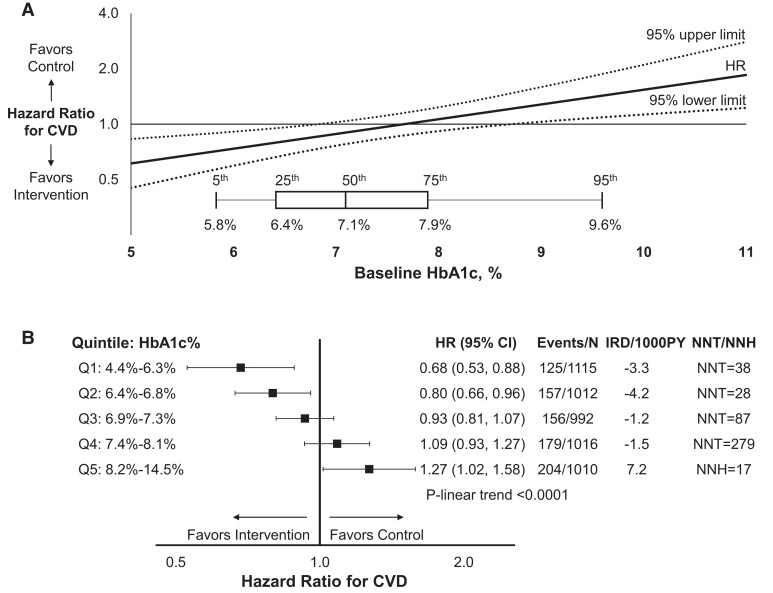
Adjusted hazard ratio (HR) and 95% confidence intervals for the association of randomization to the Look AHEAD lifestyle intervention with risk for incident cardiovascular disease according to (A) continuous baseline glycated hemoglobin (HbA1c) and (B) quintile of baseline HbA1c. Cardiovascular disease is defined as the composite outcome of death from cardiovascular causes, nonfatal myocardial infarction, nonfatal stroke, or hospitalization for angina. HRs are adjusted for study site and history of cardiovascular disease (stratified randomization), baseline HbA1c, and a product term of intervention arm and baseline HbA1c. Also presented are unadjusted incidence rate differences (IRD) per 1000 person-years and number needed to treat or harm over 9.6 years [NNT/NNH, calculated as NNT = 1/(control group cumulative incidence – intervention group cumulative incidence)]. *P* for interaction between continuous HbA1c and randomization arm <.0001.

Over a median follow-up of 9.6 years, the cumulative incidence of the primary composite CVD outcome was higher with increasing HbA1c quintile: 11.2% for Q1 (125 events, 9706 person-years), 15.5% for Q2 (157 events, 8637 person-years), 15.7% for Q3 (156 events, 8574 person-years), 17.6% for Q4 (179 events, 8564 person-years), and 20.2% for Q5 (204 events, 8370 person-years). We observed evidence for effect measure modification by continuous baseline HbA1c of the additive and multiplicative associations of randomization to ILI vs DSE with the primary CVD outcome (1 degree of freedom *P* for interaction < .0001). We did not observe evidence for effect modification of HbA1c in quadratic form (2 degrees of freedom *P* for interaction = .07, Fig. 1A and 1B). We observed a monotonic increase in risk for the composite CVD outcome associated with randomization to ILI across HbA1c quintile. The association of randomization to ILI with CVD risk changed direction at HbA1c of 7.7% (61 mmol/mol); between HbA1c 6.8% (51 mmol/mol) and 8.7% (72 mmol/mol) the ILI HR 95% CI included the null. Randomization to ILI was associated with lower risk for CVD for baseline HbA1c Q1 (HR: 0.68, 95% CI: 0.53, 0.88) and Q2 (HR: 0.80, 95% CI: 0.66, 0.96) and associated with higher risk for CVD for HbA1c Q5 (HR: 1.27, 95% CI: 1.02, 1.58), compared to DSE (Fig. 1). Randomization to ILI was not associated with CVD within baseline HbA1c Q3 and Q4. The Kaplan–Meier survival curve is illustrated in Supplementary Fig. S1 in the Supplemental Materials (17). Among HbA1c Q5, separation in CVD risk between ILI and DSE occurs at roughly 3.3 years after randomization when CVD incidence for the DSE group decelerates. Sensitivity analyses that included adjustment for demographic and clinical characteristics yielded within HbA1c quintile HR estimates for the association of ILI vs DSE with CVD similar in magnitude to the primary analysis (Supplementary Fig. S2) (17).

Similar patterns of higher CVD risk with higher HbA1c were observed for the secondary composite CVD outcomes (Supplementary Table S1) (17). For each secondary CVD outcome, randomization to ILI compared to DSE was associated with lower incidence of CVD within HbA1c Q1 to Q4 and higher incidence of CVD within HbA1c Q5. We assessed the association between randomization to ILI vs DSE and the individual CVD outcomes across HbA1c quintiles. Among HbA1c Q5, the ILI arm had a lower incidence of cardiovascular death, heart failure, and all-cause mortality but a higher incidence of myocardial infarction, angina, stroke, coronary-artery bypass grafting, and carotid endarterectomy compared to DSE (Supplementary Table S2) (17). The Kaplan–Meier survival curve for all-cause mortality showed that the DSE arm of HbA1c Q5 had the lowest survival during the intervention and that the all-cause mortality survival curves comparing DSE to ILI in HbA1c Q5 separate at year 2 (Supplementary Fig. S3) (17). During the intervention, 4% of the cohort underwent bariatric surgery, which did not appear to differ substantively by arm across baseline HbA1c (Supplementary Table S3) (17). Sensitivity analyses that excluded these individuals yielded within HbA1c quintile HR estimates for the association of ILI with CVD similar in magnitude to the primary analysis. There were 204 non-CVD deaths during follow-up. Rates of non-CVD death were highest among the DSE arm of HbA1c Q5 (Supplementary Table S4) (17). Estimates from analyses that accounted for the competing risk of non-CVD death were not materially different from the primary analysis results.

### Difference in Weight, HbA1c, and Medication Use by Intervention Arm and Baseline HbA1c

For all HbA1c quintiles, mean weight loss and HbA1c reduction was greater among individuals randomized to ILI than DSE (Supplementary Figs. S4 and S5) (17). Between-arm differences in mean weight were observed out to year 7 of the intervention for all HbA1c quintiles, though ILI participants with higher baseline HbA1c tended to lose relatively less weight compared with DSE participants. Participants randomized to ILI had significantly lower HbA1c compared to those randomized to DSE for the first 2 years of the intervention for each HbA1c quintile. Generally, use of any diabetes medication, insulins, and metformin was higher among those randomized to DSE from year 1 onward across all HbA1c quintiles. Metformin use was lower for ILI than DSE individuals during the intervention (range 3-8 years) in the lower 3 HbA1c quintiles but did not differ between ILI and DSE groups for the 2 highest HbA1c quintiles (Supplementary Fig. S6) (17). Incident use of metformin, insulins, sulfonylureas, and thiazolidinediones was higher for DSE than ILI during the first 4 years of the intervention while incident use of glucagon-like peptide-1 agonists and dipeptidyl peptidase 4 inhibitors did not differ by randomization arm (Supplementary Table S5) (17). Accounting for time-varying diabetes medication use did not impact HR estimates (Supplementary Fig. S2) (17). Over the course of the intervention, use of ACE inhibitors and statins increased for all HbA1c quintiles and intervention arms (Supplementary Figs. S7 and S8) (17). Generally, use of ACE inhibitors and statins was lower among those randomized to ILI for each HbA1c quintile. For ACE inhibitors, these between-arm differences were attenuated at year 3 of the intervention. For statins, the ILI arm had lower use of these medications compared to DSE for HbA1c Q1 (to 9 years) and Q4 (to 6 years).

### On-Intervention Weight Loss Sensitivity Analyses

Of the original cohort, 4648 attended the year 2 follow-up exam, had complete body weight data, and did not have a CVD event prior to each respective follow-up exam. Supplementary Table S6 presents randomization arm; 7% weight loss distribution at follow-up year 2; and number at risk, events, and cumulative incidence of the primary CVD endpoint by HbA1c quintile. Among individuals randomized to DSE, 12% achieved 7% weight loss of the baseline body weight at year 2, and this was consistent across HbA1c quintile (17). Among the individuals randomized to ILI, 42% achieved ≥7% weight loss of the baseline body weight at year 2, ranging from 46% in HbA1c Q1 to 31% in HbA1c quintile 5. Supplementary Fig. S9 summarizes numbers of participants who achieved the 7% weight loss goal at follow-up year 2, loss to follow-up, and incident CVD events for all 2750 individuals randomized to ILI (17). Of the individuals in the ILI arm who achieved the 7% weight loss goal at year 2, 90% (881) achieved the 7% weight loss goal at year 1 and had sustained 2-year weight loss. Supplementary Fig. S10 presents HRs for CVD when achieving 7% weight loss at year 2 vs achieving less than 7% weight loss after adjustment for baseline demographics, clinical and behavioral CVD risk factors and medications, diabetes duration and medications, and cohort attrition (17). We did not observe evidence for effect modification by baseline HbA1c of the association between achieving 7% weight loss and CVD risk (*P* for interaction = .85). HRs for CVD ranged from 0.72 (95% CI: 0.51, 1.02) for HbA1 Q1 to 0.83 (95% CI: 0.61, 1.15) for individuals who achieved 7% baseline weight loss at year 2 of the intervention compared to individuals who did not achieve the 7% weight loss goal.

## Discussion

We reanalyzed data from the Look AHEAD randomized trial of adults with type 2 diabetes and overweight or obesity and assessed the impact of baseline level of HbA1c on the association between randomization to the ILI and cardiovascular risk over 10 years. We observed a differential association between randomization to the lifestyle intervention and CVD risk by baseline HbA1c, wherein randomization to the lifestyle intervention was associated with CVD prevention at an HbA1c level below 6.8% and was associated with higher CVD risk above an HbA1c level 8.7% compared to the control group. This between-arm difference in CVD risk among those with the highest baseline HbA1c did not emerge until roughly 3 years after randomization, and the pattern of risk differed by individual CVD endpoints. ILI was associated with lower risk of CVD death but with higher risk for nonfatal CVD outcomes of myocardial infarction, stroke, and hospitalized angina among those with high baseline HbA1c; between-arm differences in risk for stroke and hospitalized angina were most pronounced of these individual CVD outcomes. Guidelines emphasize lifestyle change including weight loss and regular physical activity for individuals with type 2 diabetes and overweight or obesity, and there is observational evidence that these changes improve health outcomes (6). However, our findings show that among individuals with HbA1c ≥ 8.7% who are at a high absolute risk for cardiovascular events, the evidence-based Look AHEAD intervention was not associated with CVD prevention and was associated with a higher CVD risk. These findings identify a need to develop effective lifestyle interventions for CVD prevention targeting individuals with type 2 diabetes and high HbA1c. It may be that ILI is most effective when coupled with relatively good glycemic control.

We assessed potential explanations for the observed differential risk in CVD from ILI by baseline HbA1c. We observed that including adjustment for potential confounding factors of the HbA1c and CVD association and excluding individuals who underwent bariatric surgery during the intervention did not materially impact our findings. We observed that randomization to intensive lifestyle was associated with lower incidence of total mortality across quintiles of baseline HbA1c and that accounting for the competing risk of non-CVD death did not alter our primary findings. These findings do not provide evidence to support the hypothesis that individuals with high HbA1c who were randomized to ILI had greater underlying morbidity that placed them at higher risk compared to their DSE counterparts.

Because the lifestyle intervention was targeted to achieve weight loss, we also assessed whether our results were sensitive to achieving the goal of 7% baseline weight loss during the first 2 years, and this analysis yielded several important findings. First, just over half of individuals randomized to the lifestyle intervention were able to achieve the weight loss goal over the first year of the intervention, and less than half achieved this goal at year 2. Second, the likelihood of achieving and sustaining the weight loss goal decreased with higher baseline HbA1c among the lifestyle intervention group. Third, the highest risk for CVD in the entire trial cohort was observed among individuals at the upper end of the spectrum of baseline HbA1c who were randomized to the lifestyle intervention but were not able to achieve the weight loss goal at year 2. In contrast, the lowest risk for CVD in the cohort was observed among individuals at the high end of baseline HbA1c who were randomized to the control group and were able to achieve the weight loss goal at year 2 (≥7% of their baseline weight). Lastly, and maybe most importantly, across the spectrum of baseline HbA1c, individuals who were able to achieve the weight loss goal at year 2 had lower CVD risk than individuals who did not achieve such weight loss at year 2. Overwhelmingly, those randomized to ILI who achieved the weight loss goal at year 2 had also achieved the weight loss goal at year 1. Therefore, achieving and maintaining substantial weight loss for an extended period of time is associated with CVD prevention for individuals with type 2 diabetes and high HbA1c. This aligns with prior work in Look AHEAD that has shown the cardiovascular and mortality benefits overall among participants who achieved substantial and sustained weight loss (15, 18).

For those with high baseline HbA1c, the survival curves comparing lifestyle to control do not separate until 3 years after randomization. This delay in risk difference does not support the hypothesis that starting lifestyle would trigger acute CVD. At high HbA1c, individuals in the control group were more likely to die from CVD and non-CVD causes than those in the intervention group, starting around 2 years after randomization. Participants and their clinicians were not masked to randomization arm. It is possible that participants, particularly those with higher baseline HbA1c, who were randomized to the lifestyle group received greater attention for safety from their health care providers resulting in earlier detection of less severe CVD events. This hypothesis is supported by our findings of higher rates of hospitalization for angina but lower rates of fatal CVD and overall mortality among the lifestyle group vs control at high HbA1c.

Several methodological considerations should be discussed. First, this is a post hoc analysis that was not included in the original design of the Look AHEAD trial, and our findings are exploratory. Our results were robust in several sensitivity analyses. Second, Look AHEAD participants may have sought off-study weight loss and treatment strategies. Third, we based our primary effect modification analyses on a single measurement of HbA1c, though a strength of HbA1c is that it has very low variability over the short term compared to other measures of glucose. Fourth, these results may be generalizable to only a subsample of individuals with type 2 diabetes with a higher burden of obesity and hypertension and more favorable lipid and glucose profiles than the broader US population with diabetes (10). Last, HbA1c may be a surrogate marker for some other morbidity or health determinant for which the lifestyle intervention differs in CVD prevention. Measures of insulin sensitivity were not captured in Look AHEAD, and these data may be important to subsequent analyses.

Our findings demonstrate that, among individuals with type 2 diabetes, overweight or obesity, and HbA1c below 6.8%, ILI is associated with lower risk for cardiovascular disease over 10 years. These findings also identify an important question: the potential differential effect of lifestyle interventions for cardiovascular disease prevention among individuals with type 2 diabetes and high HbA1c. It is unclear why ILI failed for CVD prevention for individuals in Look AHEAD with higher HbA1c. Among US adults with diagnosed diabetes, 1 in 4 have an HbA1c ≥ 8.0%, and research is needed to understand how to develop and tailor lifestyle interventions to be successful for this large population subgroup (19).

## Data Availability

The data used for analysis during the current study were housed at the data coordinating center and are not available for public distribution. However, all data used for this analysis were supplied by Look AHEAD investigators to the National Institute of Diabetes and Digestive and Kidney Diseases Central Repository and are publicly available at https://repository.niddk.nih.gov/studies/look-ahead/? query = Look%20AHEAD.

## Clinical Trial Information

This trial is registered at Clinicaltrials.gov: https://clinicaltrials.gov/ct2/show/NCT00017953?term=NCT00017953&draw=2&rank=1, Identifier: NCT00017953.
